# Algorithm for heart rate extraction in a novel wearable acoustic sensor

**DOI:** 10.1049/htl.2014.0095

**Published:** 2015-02-24

**Authors:** Guangwei Chen, Syed Anas Imtiaz, Eduardo Aguilar–Pelaez, Esther Rodriguez–Villegas

**Affiliations:** Department of Electrical and Electronic Engineering, Imperial College London, London SW7 2AZ, UK

**Keywords:** phonocardiography, feature extraction, body sensor networks, acoustic transducers, biomedical transducers, medical signal processing, acoustic signal processing, pneumodynamics, patient monitoring, data acquisition, signal classification, heart rate extraction algorithm, novel wearable acoustic sensor, phonocardiography, heart sound listening, cardiac abnormalities, heart cycle, acoustic signal acquisition, S1 heart sound detection, S2 heart sound detection, heart rate extraction, signal acquisition, commercial devices, data acquisition, dataset, acoustic heart sound classification, breathing monitoring, long-term wearable vital signs monitoring

## Abstract

Phonocardiography is a widely used method of listening to the heart sounds and indicating the presence of cardiac abnormalities. Each heart cycle consists of two major sounds – *S*1 and *S*2 – that can be used to determine the heart rate. The conventional method of acoustic signal acquisition involves placing the sound sensor at the chest where this sound is most audible. Presented is a novel algorithm for the detection of *S*1 and *S*2 heart sounds and the use of them to extract the heart rate from signals acquired by a small sensor placed at the neck. This algorithm achieves an accuracy of 90.73 and 90.69%, with respect to heart rate value provided by two commercial devices, evaluated on more than 38 h of data acquired from ten different subjects during sleep in a pilot clinical study. This is the largest dataset for acoustic heart sound classification and heart rate extraction in the literature to date. The algorithm in this study used signals from a sensor designed to monitor breathing. This shows that the same sensor and signal can be used to monitor both breathing and heart rate, making it highly useful for long-term wearable vital signs monitoring.

## 1 Introduction

Heart sounds have been a source of information for diagnosis of patients’ conditions since the late 19th century via the use of the stethoscope [1]. Trained doctors can listen for abnormal heart sounds in what is commonly referred to as cardiac auscultation. The conventional method of analysing heart sounds is known as phonocardiography (PCG) where a microphone, normally placed on the chest, is used to record the sounds, which can be analysed by a doctor. Each heart cycle consists of two major sounds: *S*1 followed by *S*2. Other sounds and murmurs can indicate abnormalities. The distance between two *S*1 sounds is the duration of one heart cycle that can be used to determine the heart rate.

PCG has been used broadly for diagnosis of certain cardiac conditions and, in the later part of the 20th century, has received attention by the engineering community with the goal of investigating signal processing techniques to achieve automatic segmentation and marking of PCG signals. The capability of segmenting heart sound into heart cycles and distinguishing between cardiac phases, by appropriately detecting the first and second heart sound, is useful since it can be used to calculate the heart rate.

Several research groups have used different signal processing techniques for the segmentation of the two main heart sounds – *S*1 and *S*2 – from PCG signals for different applications including the evaluation of heart rate. These can be broadly divided into two categories [2]. First, those that use ECG as a reference for synchronisation of heart cycles and, second, those that rely solely on the PCG signal without any reference. The latter approach is appropriate for wearable devices since it relies on smaller number of sensors. This Section briefly reviews some of these techniques that do not require ECG reference and reports their accuracy.

Liang *et al.* [3] presented a method for heart sound segmentation by detecting peaks from the normalised average Shannon energy of the low-pass filtered input signal. They tested the algorithm using 515 cardiac cycles obtained from 37 subjects and reported sensitivities of 93 and 84% on clean and normal signals, respectively. They further improved their algorithm's performance using wavelet decomposition [4] instead of low-pass filtering, with sensitivities of 96.7 and 93% on clean and normal signals. Brusco and Nazeran [5] presented an algorithm, also using peaks from the normalised Shannon energy, for the classification of different heart sounds. For the segmentations of *S*1 and *S*2 sounds, they used a threshold to classify the peaks and considered the distances between them. They achieved an overall accuracy of 79.3% for the detection of heart cycles. In another method, Kumar *et al.* [2] used wavelet decomposition of the input signal to extract high-frequency components. They used Shannon energy of these components for classification of *S*1 and *S*2 sounds and estimation of heart rate. Their dataset consisted of a maximum of 110 min of data recorded from 55 patients with 7530 heart cycles and achieved a sensitivity of 97.95%. Wang *et al.* [6] also used Shannon energy of the signal in a multistage method for the segmentation of *S*1 sounds. They first used wavelet transform to isolate potential *S*1 and *S*2 sounds followed by detection of *S*1 using Shannon energy. They reported sensitivity of 93.2% with test data consisting of 207 heart cycles.

Gamero and Watrous [7] employed a statistical approach using hidden Markov model (HMM) for the classification of *S*1 and *S*2 sounds. Their dataset included 20 s recording each from 80 subjects and their algorithm achieved a sensitivity of 95%. Ricke *et al.* [8] also used HMM after computing the Shannon energy of the input signal. They reported a sensitivity of 98% on a test set that consisted of 2286 s of clean (noise free) data. Using wavelet decomposition and HMM, Lima and Barbosa [9] reported 99.1% sensitivity for the detection of *S*2 sounds from 700 heart cycles.

Ari *et al.* [10] presented a method in which the PCG signal is first low-pass filtered with a cut-off frequency of 150 Hz. The energy peaks from the filtered signal are extracted using a varying threshold and are then classified in an iterative process involving time search and amplitude threshold reduction. They reported the algorithm's accuracy as 97.5% using a test set with 357 heart cycles. Yamacli *et al.* [11] performed wavelet decomposition of the normalised input signal followed by moving window integration of the squared (energy) signal. The energy peaks are then detected by a varying threshold which are classified as *S*1 or *S*2 based on time conditions. With 326 heart cycles from 53 patients they reported sensitivities of 91.47 and 88.95% for *S*1 and *S*2 classifications, respectively. Gupta *et al.* [12] used wavelet features with a grow and learn algorithm to successfully segment 90.29% of 340 heart cycles with murmurs. Finally, Chen *et al.* [13] presented a PCG-based heart rate measurement method using template extraction and matching of the filtered input signal. They used three subjects for testing and reported a root mean square (RMS) error value in the calculation of heart rate as 2.4 bpm with the subjects in resting position.

In all of the methods above, the sensor to record heart sounds was placed on the chest. Most of these sensors were either bulky or required strapping around the chest, which adds to the discomfort of the user.

Popov *et al.* [14] used a different approach involving a piezoelectric sensor placed on the throat to acquire carotid pulse sounds. They applied autocorrelation analysis to 20 s recording sections of band-pass filtered input signal for the estimation of heart rate. They used 10 min recordings from eight subjects during treadmill exercise and achieved a standard deviation (SD) of 3.4 bpm. However, the bias of the regression estimation is large. For a heart rate of 60 bpm, the bias would be +11.75 bpm.

**Figure 1:**
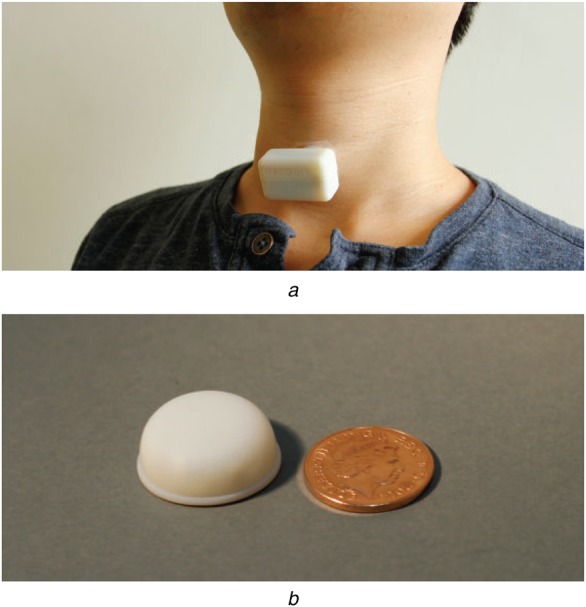
Sensor used to acquire signals *a* Acoustic sensor being worn by subject on neck *b* Second generation of sensor with smaller size (compared to two pence coin)

In our prior work, we used a wearable sensor placed at the suprasternal notch to monitor breathing [15]. The sensor, shown in Fig. 1, acquired signals that also included heart sounds. For the detection of respiratory rate, heart sounds are considered as interference and need to be removed. However, once localised, these can be used to detect the *S*1 and *S*2, and subsequently the heart rate.

The signals acquired at the suprasternal notch are intrinsically different to those observed at the surface of the chest. Signals measured at the chest have travelled a short distance propagating from the heart, through lung tissue and finally through muscle and bone. This allows for the signal to be less filtered and have higher-frequency components. Signals measured at the suprasternal notch have travelled a greater distance from the heart and principally propagated along the arterial wall of the carotid artery. As a result, the signals are of similar timing characteristics but of significantly lower bandwidth. However, the use of one small sensor to perform the dual role of respiratory and heart rate detection is advantageous since it obviates the need for an additional sensor, thus making it more comfortable for the subjects undergoing long-term monitoring.

In this Letter, we present a novel algorithm for the detection of heart rate from heart sounds acquired from a sensor placed at the suprasternal notch, originally designed to monitor breathing. Since the sensor was used to monitor breathing, the heart sound signals are much attenuated and are ‘corrupted’ with respiratory signals. The idea of the algorithm described in this Letter is to recover the heart sound signals from the respiratory signal, and then evaluate the heart rate. Section 2 explains the different stages of this algorithm in detail. Section 3 describes the dataset of over 38 h of acoustic signals used to test the algorithm. The performance of the algorithm for the calculation of heart rate is presented in Section 3 and further discussed with conclusions in Section 4.

## 2 Algorithm

**Figure 2:**
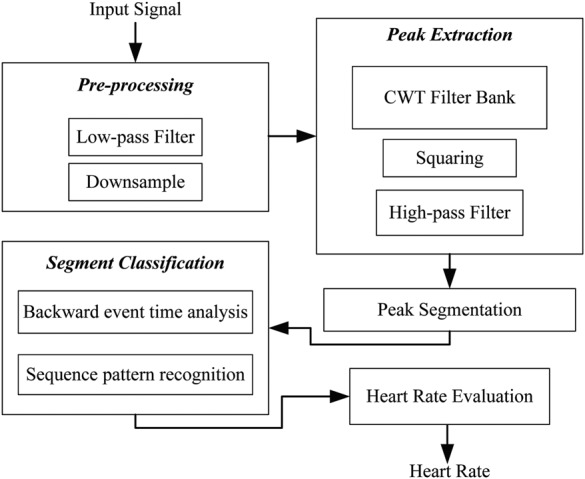
Block diagram of proposed algorithm showing all processing stages

In this Section, a novel algorithm for the detection of heart rate is presented. The input acoustic signal is first filtered to be in the frequency of interest. It is analysed with a continuous wavelet transform (CWT)-based filter bank to extract peak frequencies that can be potential *S*1 and *S*2 sounds. The peaks are later grouped together and classified with a dynamic detection threshold using a set of rules to identify *S*1 and *S*2 events. A block diagram of the proposed algorithm is shown in Fig. 2 and the details of each processing stage are given below.

### 2.1 Pre-processing

**Figure 3:**
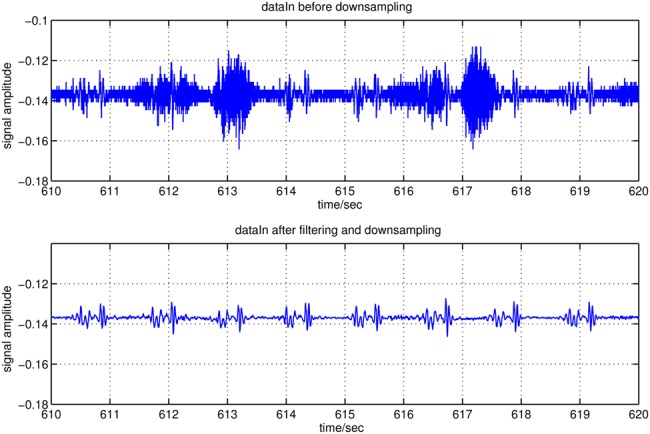
Example of input signal section before and after filtering with an eighth-order LPF at 100 Hz and downsampling from 2205 to 220.5 Hz

The input signal, sampled at 2205 Hz using a 10 bit analogue-to-digital converter (ADC), is initially filtered with an eighth-order low-pass filter with a cut-off frequency of 100 Hz. The cut-off frequency is chosen based on the signal characteristics. The filtered signal is subsequently downsampled by a factor of 10 to reduce data rate for reduction of unnecessary computational complexity because of the very high oversampling of heart sounds (originally meant for breathing sounds). As a result, the new sampling frequency is 220.5 Hz. An example of the input signal before and after filtering and downsampling is shown in Fig. 3.

### 2.2 Peak extraction

For the heart sounds to be detected, the peaks of energy in the time–frequency plane need to be located. Owing to the variation in peak frequencies between *S*1 and *S*2 sounds, as well as between different subjects, a filter bank approach provides better resolution than a broader single band-pass filter approach encompassing the region where energy peaks are expected. The specifications required of this filter bank are particular in that both time resolution and frequency resolution are important. In other words, the bandwidth of each filter needs to be as narrow as possible while still guaranteeing a short enough impulse response.

The filter bank is realised using CWT filters with Meyer mother wavelet. The centre frequencies of the filter bank were chosen to be a spread of 15 filters ranging from 8.9 Hz all the way until 23 Hz, focusing on the frequency region where peaks are expected to occur. The output of the CWT filter can have both positive and negative values, given that the intrinsic transformation performed is a convolution in time of the signal and the mother wavelet. Thus, the output is squared so that the peaks on both positive and negative sides of the signal can be utilised.

The filter bank covers a selective frequency range which extends further than the normal bandwidth of single heart sounds *S*1 and *S*2. Furthermore, heart sounds can have varied peak frequencies between different subjects. Thus, the highest squared value output of the CWT filter bank for each sample was selected to allow for such inter-subject variations. In some cases, high amplitude artefacts can maximise the value of the CWT filter bank output. However, these artefacts, resulting from loud breathing, snoring or speaking have a longer duration than heart sounds (longer than 200 ms) and can be discriminated later in the algorithm.

A high-pass filtering is performed on the output of the filter bank for better selection of transient-like signals once the peaks have been extracted. This is achieved using an eighth-order finite impulse response filter with a cut-off frequency of 40 Hz.

### 2.3 Signal grouping/segmentation and classification

The extracted peaks are then verified using an amplitude threshold that varies dynamically along time as a function of the input signal. Multiple peaks that are above the threshold and separated by <100 ms are grouped together in one segment. The duration of the segment is then defined by it starting at the first part of the signal above the threshold and finishing at the last, encompassing all the samples in between.

The detection threshold starts off at a given value but obtains updated to a proportional value of the peak amplitude – see (1) – when a segment is classified as heart sound following the normal expected time conditions in the later blocks of the signal processing. The coefficients *c*_1_ and *c*_2_ are defined to be 0.9 and 0.1, respectively, whilst the ratio factor *r*_1_ is set to (1/3). If there is a long section where all values are above or below the threshold, then it is reset to initial value. This long section is the time in which five *S*1 sounds are expected

(1) $${\rm thr}_{{\rm new}} = c_1 \times {\rm thr}_{{\rm old}} + c_2 \times r_1 \times {\rm peakamp}\lpar n\rpar \eqno\lpar 1\rpar $$

Once the segments are identified, they are classified as either *S*1 or *S*2 using a series of time-based rules.

### 2.4 Segment classification

This stage consists of a set of conditions that are executed sequentially if the previous one fails. These rules have been divided into two different categories. The first category, ‘backward event time analysis’, covers a set of three normal scenarios and four exceptions for classification of a given segment. The second category, ‘sequence pattern recognition’, is triggered when the last five segments fall within a certain time pattern. All of these rules are explained in detail below.

#### 2.4.1 Backward event time analysis

This method consists of detecting *S*1 and *S*2 heart sounds based on the time separation between detected peak segments. It is based on the time separation between peak segments in comparison to two time variables: the time separating an *S*1 heart sound and its corresponding *S*2 (*D*1); and the time difference between two *S*1 sounds (*D*2), which is equivalent to one heart cycle period.

The algorithm starts with *D*1 and *D*2 defined as in (2) and updates them dynamically as the algorithm interprets more data (based on conditions below). The *D*1–*D*2 ratio is calculated based on the relationship presented by Weissler *et al.* [16].

For the purpose of detecting peaks when variations in *D*1 and *D*2 occur, the margin ratios *k*1 and *k*2, as described in (3), are introduced. These allow for peaks occurring slightly earlier or later (−15 and +10%) with respect to the latest *S*1 heart sound to be considered in the backward event time analysis. In the following rules, the segment classification at *n* is denoted by sc(*n*), while the segment time is denoted by st(*n*)

(2) $$D1 = 0.32\, {\rm s}\quad D2 = 0.87\, {\rm s}\eqno\lpar 2\rpar $$

(3) $$k1 = 0.85\quad k2 = 1.1\eqno\lpar 3\rpar $$

Scenario 1 This condition checks for the presence of a previously defined *S*1 at the antepenultimate segment sc(*n* − 2) to define the current segment sc(*n*) as *S*1. If an *S*1 exists at *n* − 2, the condition is passed if the distance between the segments is within the expected margins. Otherwise, the segment is labelled as undefined or ‘do not know’ (DK)

(4) $${\rm sc}\lpar n\rpar = S1\comma \; \, \, {\rm if}\left\{{\matrix{ {{\rm sc}\lpar n - 2\rpar = S1} \hfill \cr {{\rm st}\lpar n\rpar - {\rm st}\lpar n - 2\rpar \gt k1 \times D2} \hfill \cr {{\rm st}\lpar n\rpar - {\rm st}\lpar n - 2\rpar \lt k2 \times D2} \hfill \cr } } \right.\eqno\lpar 4\rpar $$

If this condition is passed, the time distance *D*2 is updated as a weighted average between the newly measured time and its previous value as shown below

(5) $$D2 = 0.9 \times D2 + 0.1 \times \lpar {\rm st}\lpar n\rpar - {\rm st}\lpar n - 2\rpar \rpar \eqno\lpar 5\rpar $$

Scenario 2 This condition looks for the presence of an *S*1 at the previous segment sc(*n* − 1) in order to define the peak at *n* as *S*2 if the time distance to the previous segment is within the *D*1 time separation expected (*S*1 to *S*2 time)

(6) $${\rm sc}\lpar n\rpar = S2\comma \; \, \, {\rm if}\left\{{\matrix{ {{\rm sc}\lpar n\rpar = {\rm DK}} \hfill \cr {{\rm sc}\lpar n - 1\rpar = S1} \hfill \cr {{\rm st}\lpar n\rpar - {\rm st}\lpar n - 1\rpar \gt k1 \times D1} \hfill \cr {{\rm st}\lpar n\rpar - {\rm st}\lpar n - 1\rpar \lt k2 \times D1} \hfill \cr } } \right.\eqno\lpar 6\rpar $$

If this condition is evaluated to be true, the time distance *D*1 is redefined as a weighted average between the newly measured time and the previous value

(7) $$D1 = 0.9 \times D1 + 0.1 \times \lpar {\rm st}\lpar n\rpar - {\rm st}\lpar n - 1\rpar \rpar \eqno\lpar 7\rpar $$

Scenario 3 This condition is similar to the previous case and looks for the presence of an *S*2 at *n* − 1 to define the segment at *n* as *S*1 if the distance between the present and the penultimate segment is that expected between an *S*2 and an *S*1

(8) $${\rm sc}\lpar n\rpar = S1\comma \; \, \, {\rm if}\left\{{\matrix{ {{\rm sc}\lpar n\rpar = {\rm DK}} \hfill \cr {{\rm sc}\lpar n - 1\rpar = S2} \hfill \cr {{\rm st}\lpar n\rpar - {\rm st}\lpar n - 1\rpar \gt k1 \times \lpar D2 - D1\rpar } \hfill \cr {{\rm st}\lpar n\rpar - {\rm st}\lpar n - 1\rpar \lt k2 \times \lpar D2 - D1\rpar } \hfill \cr } } \right.\eqno\lpar 8\rpar $$

In this condition, there is no redefinition of *D*1 or *D*2 because the analysed time corresponds to *D*1–*D*2, which is a measure between two different heart cycles, not directly correlated to either the periodicity of neuromuscular excitation of the heart (*D*1) or the heart cycle event sequence between and separation between two of its sounds (*D*2).

If the three ‘normal scenarios’ fail to classify a segment, it is labelled as DK. If two consecutive segments are labelled as DK, then a further series of ‘exceptions’ are triggered to attempt and define the current segment.

Exceptions 1 and 2 These are two similar conditions (for *S*1 and *S*2, respectively) look for a DK at *n* − 1 to define the peak at *n* as *S*1 or *S*2 based on what was defined at *n* − 2 if the separation between the *n* − 2 and *n* peaks is within the *D*2 margins, i.e., the one between an *S*1 and an *S*1 or an *S*2 and an *S*2

(9) $${\rm sc}\lpar n\rpar = S1\, \, {\rm or}\, \, S2\comma \; \, \, {\rm if}\left\{{\matrix{ {{\rm sc}\lpar n\rpar = {\rm DK}} \hfill \cr {{\rm sc}\lpar n - 1\rpar = {\rm DK}} \hfill \cr {{\rm sc}\lpar n - 2\rpar = S1\, \, {\rm or}\, \, S2\comma \; \, \, {\rm respectively}} \hfill \cr {{\rm st}\lpar n\rpar - {\rm st}\lpar n - 2\rpar \gt k1 \times D2} \hfill \cr {{\rm st}\lpar n\rpar - {\rm st}\lpar n - 2\rpar \lt k2 \times D2} \hfill \cr } } \right.\eqno\lpar 9\rpar $$

If the segment at *n* − 2 was an *S*1, then *D*2 is updated using (5) as in condition Scenario 1.

Exception 3 This condition is used to define the current segment as *S*1 if the time distance to segment *n* − 2 is *D*2 × 2 − *D*1 and the segment at *n* − 2 had previously been classified as *S*2. In this case, the segment at *n* − 1 is left as DK

(10) $${\rm sc}\lpar n\rpar = S1\comma \; \, \, {\rm if}\left\{{\matrix{ {{\rm sc}\lpar n\rpar = {\rm DK}} \hfill \cr {{\rm sc}\lpar n - 1\rpar = {\rm DK}} \hfill \cr {{\rm sc}\lpar n - 2\rpar = S2} \hfill \cr {{\rm st}\lpar n\rpar - {\rm st}\lpar n - 2\rpar \gt \lpar k1 \times D2\rpar \times 2 - D1} \hfill \cr {{\rm st}\lpar n\rpar - {\rm st}\lpar n - 2\rpar \lt \lpar k2 \times D2\rpar \times 2 - D1} \hfill \cr } } \right.\eqno\lpar 10\rpar $$

Exception 4 This condition defines the current segment as *S*1 if the time distance to segment *n* − 2 is *D*2 − *D*1 and the segment at *n* − 2 had previously been classified as *S*2. In this case, as in Exception 3, the segment at *n* − 1 is left as DK

(11) $${\rm sc}\lpar n\rpar = S1\comma \; \, \, {\rm if}\left\{{\matrix{ {{\rm sc}\lpar n\rpar = {\rm DK}} \hfill \cr {{\rm sc}\lpar n - 1\rpar = {\rm DK}} \hfill \cr {{\rm sc}\lpar n - 2\rpar = S2} \hfill \cr {{\rm st}\lpar n\rpar - {\rm st}\lpar n - 2\rpar \gt \lpar k1 \times D2\rpar - D1} \hfill \cr {{\rm st}\lpar n\rpar - {\rm st}\lpar n - 2\rpar \lt \lpar k2 \times D2\rpar - D1} \hfill \cr } } \right.\eqno\lpar 11\rpar $$

In the event that all ‘exceptions’ fail to determine whether a segment is *S*1 or *S*2, it is left as DK.

#### 2.4.2 Sequence pattern recognition

The pattern recognition strategy has been designed to provide the least possible false *S*1 and *S*2 detections. The last five peak segments must fall within particular time location restrictions in order for a pattern to be detected and considered as correct *S*1 and *S*2 heart sounds. This is useful at the start of the classification when the algorithm is initialised and after any discontinuity in peaks that could not be dealt with by any of the ‘scenarios’ and ‘exception’ conditions

(12) $$\eqalign{& {\rm sc}\lpar n - 4\colon n\rpar = \lsqb S2\comma \; \, S1\comma \; \, S2\comma \; \, S1\comma \; \, S2\rsqb \comma \; \, \cr & {\rm if}\left\{{\matrix{ {{\rm st}\lpar n\rpar - {\rm st}\lpar n - 1\rpar \gt k1 \times D1} \hfill \cr {{\rm st}\lpar n\rpar - {\rm st}\lpar n - 1\rpar \lt k2 \times D1} \hfill \cr {{\rm st}\lpar n\rpar - {\rm st}\lpar n - 2\rpar \gt k1 \times D2} \hfill \cr {{\rm st}\lpar n\rpar - {\rm st}\lpar n - 2\rpar \lt k2 \times D2} \hfill \cr {{\rm st}\lpar n\rpar - {\rm st}\lpar n - 3\rpar \gt k1 \times D1 + D2} \hfill \cr {{\rm st}\lpar n\rpar - {\rm st}\lpar n - 3\rpar \lt k2 \times D1 + D2} \hfill \cr {{\rm st}\lpar n\rpar - {\rm st}\lpar n - 4\rpar \gt k1 \times D2 + D2} \hfill \cr {{\rm st}\lpar n\rpar - {\rm st}\lpar n - 4\rpar \lt k2 \times D2 + D2} \hfill \cr } } \right.} \eqno\lpar 12\rpar $$

Patterns 1 and 2 These two patterns, defined in (12) and (13), respectively, look for cases where the preceding four segments have not been classified but happen to follow a time separation pattern with the present segment that coincides with that expected based on the time separations *D*1 and *D*2 at that particular point in time. It is important to remember that the time separations *D*1 and *D*2 are dynamic values that are updated as candidates *S*1 and *S*2 events are detected

(13) $$\eqalign{& {\rm sc}\lpar n - 4\colon n\rpar = \lsqb S1\comma \; \, S2\comma \; \, S1\comma \; \, S2\comma \; \, S1\rsqb \comma \; \cr & \, {\rm if}\left\{{\matrix{ {{\rm st}\lpar n\rpar - {\rm st}\lpar n - 1\rpar } \hfill & \matrix{ \gt k1 \times D2 - D1 \hfill \cr \lt k2 \times D2 - D1 \hfill} \hfill \cr {{\rm st}\lpar n\rpar - {\rm st}\lpar n - 2\rpar } \hfill & \matrix{ \gt k1 \times D2 \hfill \cr \lt k2 \times D2 \hfill} \hfill \cr {{\rm st}\lpar n\rpar - {\rm st}\lpar n - 3\rpar } \hfill & \matrix{ \gt k1\lpar D2 - D1\rpar + D2 \hfill \cr \lt k2\lpar D2 - D1\rpar + D2 \hfill} \hfill \cr {{\rm st}\lpar n\rpar - {\rm st}\lpar n - 4\rpar } \hfill & \matrix{ \gt k1 \times D2 + D2 \hfill \cr \lt k2 \times D2 + D2 \hfill} \hfill \cr } } \right.} \eqno\lpar 13\rpar $$

Pattern 3 This condition consists of detecting a pattern where there is some peak time separation repetition similar to that expected from *S*1 and *S*2 sounds but where this time separation recognition is not limited by the *D*1 and *D*2 bounds presented above. To detect a new pattern without these bounds, a new *D*2 is defined as the time separation between the present segment at *n* and the second preceding one (*n* − 2). This way, regardless of whether the present segment is *S*1 or *S*2, the time difference between the two is taken as being one heart cycle duration (14)

(14) $$D2_x = {\rm st}\lpar n\rpar - {\rm st}\lpar n - 2\rpar \eqno\lpar 14\rpar $$

For Pattern 3 to be evaluated, the newly defined *D*2 separation (*D*2*_x_*) needs to pass one further condition. This is based on the expected limit of heart rate variation and maximum heart rate for pattern recognition (HR_max_ = 200 bpm). Equation (15) defines the upper limit of heart rate variability (increase) which has been defined considerably high so as to only remove the cases that are clearly above the expected ranges of HR increase and to allow for enough dynamic variation

(15) $${\rm HR}_{{\rm var}\, \, {\rm limit}} = 3.77 \times \lpar {\rm seconds}\, \, {\rm since}\, \, {\rm last}\, \, D2\, \, {\rm update}\rpar + 17\eqno\lpar 15\rpar $$

(16) $${\rm HR}_{{\rm measured}\, \, {\rm var}} = \displaystyle{{60} \over {D2_x }}\, - \, \displaystyle{{60} \over {D2_{{\rm last}} }}\eqno\lpar 16\rpar $$

The condition for carrying out Pattern 3 evaluation is defined in (17)

(17) $$\eqalign{& {\rm conditions}\, \, {\rm for}\, \, {\rm testing}\, \, {\rm pattern}\, \, 3 \cr & = \left\{{\matrix{ {{\rm HR}_{{\rm measured}\, \, {\rm var}} \lt {\rm HR}_{{\rm var}\, \, {\rm limit}} } \hfill \cr {\displaystyle{{60} \over {D2_x }} \lt {\rm HR}_{\max } } \hfill \cr } } \right.} \eqno\lpar 17\rpar $$

Once these conditions are met, a new value of *D*1 is defined based on the correlation presented in [16], shown in (18)

(18) $$D1_x = - 0.0018 \times \displaystyle{{60} \over {D2_x }} + 0.456\eqno\lpar 18\rpar $$

(19) $$\eqalign{& {\rm sc}\lpar n - 4\colon n\rpar = \lsqb S2\comma \; \, S1\comma \; \, S2\comma \; \, S1\comma \; \, S2\rsqb \comma \; \cr & \, {\rm if}\left\{{\matrix{ {{\rm st}\lpar n\rpar - {\rm st}\lpar n - 1\rpar } \hfill & \matrix{ \gt k1 \times D1_x \hfill \cr \lt k2 \times D1_x \hfill} \hfill \cr {{\rm st}\lpar n\rpar - {\rm st}\lpar n - 3\rpar } \hfill & \matrix{ \gt k1 \times D1_x + D2_x \hfill \cr \lt k2 \times D1_x + D2_x \hfill} \hfill \cr {{\rm st}\lpar n\rpar - {\rm st}\lpar n - 4\rpar } \hfill & \matrix{ \gt k1 \times D2_x + D2_x \hfill \cr \lt k2 \times D2_x + D2_x \hfill} \hfill \cr } } \right.} \eqno\lpar 19\rpar $$

Pattern 4 This condition is effectively the same as Pattern 3, but with the exception that *D*1*_x_* is defined as the last value of *D*1.

Pattern 5 This condition looks for time separations that would have been caused by *S*1 sounds, and hence separated by their respective *D*2. It also needs to pass the same conditions of heart rate variability and maximum heart rate as expressed in (17). The new *D*2 is defined based on the separation of the last two segments (20)

(20) $$D2_x = {\rm st}\lpar n\rpar - {\rm st}\lpar n - 1\rpar \eqno\lpar 20\rpar $$

(21) $$\eqalign{& {\rm sc}\lpar n - 4\colon n\rpar = \lsqb S1\comma \; \, S1\comma \; \, S1\comma \; \, S1\comma \; \, S1\rsqb \comma \; \cr & \, {\rm if}\left\{{\matrix{ {{\rm st}\lpar n - 1\rpar - {\rm st}\lpar n - 2\rpar } \hfill & \matrix{ \gt k1 \times D2_x \hfill \cr \lt k2 \times D2_x \hfill} \hfill \cr {{\rm st}\lpar n - 2\rpar - {\rm st}\lpar n - 3\rpar } \hfill & \matrix{ \gt k1 \times D2_x \hfill \cr \lt k2 \times D2_x \hfill} \hfill \cr {{\rm st}\lpar n - 3\rpar - {\rm st}\lpar n - 4\rpar } \hfill & \matrix{ \gt k1 \times D2_x \hfill \cr \lt k2 \times D2_x \hfill} \hfill \cr } } \right.} \eqno\lpar 21\rpar $$

### 2.5 Heart rate calculation

For the heart rate to be calculated based on the classification of segments as *S*1 or *S*2, heartbeat cycles need to be detected. Therefore any *S*2 following an *S*1 is merged to the corresponding *S*1 so as to form a single entity representing a single heartbeat. The number of heartbeats detected in a particular ‘time’ interval is then used to calculate the heart rate using (22)

(22) $${\rm heart}\, \, {\rm rate} = \displaystyle{{{\rm number}\, \, {\rm of}\, \, {\rm intervals}} \over {{\rm time}\lpar {\rm s}\rpar }} \times 60\eqno\lpar 22\rpar $$

## 3 Performance analysis

### 3.1 Database

Data were obtained as part of a clinical study that was conducted in a sleep study room of the National Hospital for Neurology and Neurosurgery (UK). The study was approved by the Medicine and Healthcare Products Regulatory Agency and the Research Ethics Committee of the UK National Hospital for Neurology and Neurosurgery. A wireless acoustic sensor was placed at the suprasternal notch during night time which sampled data at a frequency of 2205 Hz and transmitted to a nearby base station for further analysis.

**Figure 4:**
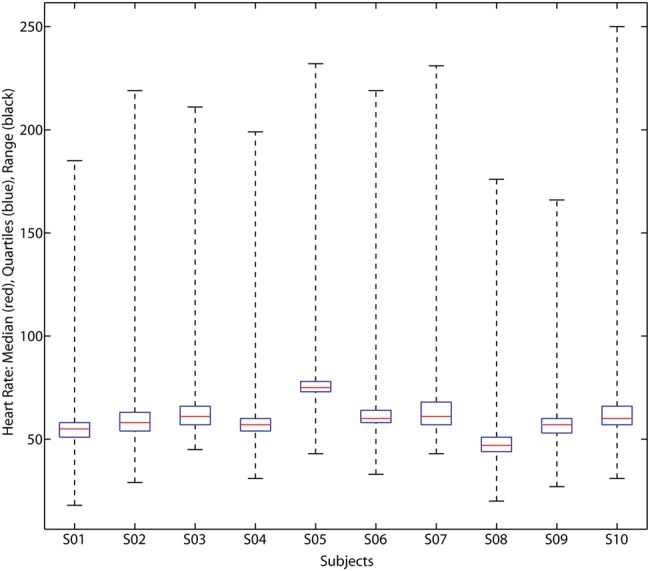
Heart rate variation and ranges in each subject as recorded by the reference device

At the same time, two external devices were used to compute reference heart rate for performance evaluation: SOMNOscreen by SomnoMedics [17] and PULSOX 300i pulse oximeter by Konica-Minolta [18]. The SomnoMedics device provides a pulse output calculated based on the photoplethysmography signal which is used by a software to calculate the heart rate. The Konica-Minolta pulse oximeter also provides its own heart rate numerical output along with the oxygen saturation based on its own photoplethysmography sensor. Data from all three sensors was synchronised at the end of each recording using a single reference clock and a total of over 38 h of data recorded during sleep from ten different subjects was evaluated. The heart rate varied differently for each subject throughout the night. The range of variation and the median heart rate for all subjects is shown in Fig. 4.

### 3.2 Results

The proposed algorithm computes heart rate in a window of 60 s. This was compared with the values obtained from two commercial monitors: SomnoMedics and Konica-Minolta. They have a sampling rate of 4 and 1 Hz, respectively, therefore the average value over 60 s from these sensors was used to compare the output from the algorithm.

The numerical difference of the values calculated by the algorithm and those provided by the two external devices have been characterised by the quartile divisions of the difference spread. The results show that, in most cases, the algorithm achieves a high concentration of outcomes very close to zero difference with short separation between quartiles. The median difference for all subjects in both cases is < 0.5 bpm, except for S07 where the greater than normal spread is attributed to sustained presence of snoring throughout the night – even if not always saturating the ADC – and the resulting necessity for the algorithm to continuously look for patterns. The reason for this is that after a large snore, which interrupts the classification of heart sounds via the more robust ‘scenarios’ and ‘exception’ conditions, the algorithm needs to restart the classification by finding a pattern, which is more prone to errors under bad signal conditions.

To determine the overall accuracy of the algorithm, the percentage of heart rate output values that fit within a narrow error margin of *±*10% with respect to the values provided by the gold standard reference devices was calculated. The results of the algorithm expressed in this performance metric for each subject and in comparison to each reference device are shown in Table 1. It can be noted from this that the algorithm achieved results above 90% for six of the ten subjects (above 85% for nine subjects) and that the lowest value was from subject S07 for reasons explained above. The overall weighted accuracy of the algorithm is 90.73 and 90.69% with respect to the Konica-Minolta and SomnoMedics devices, respectively, based on the duration of data per subject.

**Table 1:** Percentage accuracy of algorithm with respect to Konica-Minolta and SomnoMedics devices for each subject and their weighted average

Subject	Konica-Minolta, %	SomnoMedics, %
*S*01	97.53	97.37
*S*02	91.97	89.5
*S*03	88.32	89.07
*S*04	95.53	96.06
*S*05	90.88	91.45
*S*06	94.15	93.6
*S*07	71.69	73.34
*S*08	86.53	86.26
*S*09	94.56	94.62
*S*10	93.69	93.84
weighted average	90.73	90.69

## 4 Discussion

An algorithm for the segmentation of heart sounds (*S*1 and *S*2) and extraction of heart rate from signals recorded at suprasternal notch is presented in this Letter. The performance of this algorithm has been evaluated on over 38 h of data acquired from ten different subjects during sleep in the clinical trial setting. To the best of authors’ knowledge, this represents the largest dataset a heart sound classification and heart rate extraction algorithm has been tested on. Although other studies (such as [3, 4, 11]) used data from a greater number of subjects, their total duration of data and the number of heart cycles was significantly smaller.

It is difficult to directly compare the results of this algorithm with existing methods in literature since most algorithms use signals extracted from the chest region for heart sound segmentation. Table 2 shows the performance of several heart sound segmentation algorithms that were discussed in Section 1, their test data size and number of subjects used in the study. In all these cases, the sensor for data acquisition was placed on the chest, except in this Letter and [14], where it was placed on the neck.

**Table 2:** Comparison of dataset size and results obtained in this algorithm with other works in the literature

Reference	Test data	Subjects	Results
[3]	515 cycles	37	93 and 84%
[4]	1165 cycles	77	96.7 and 92.9%
[5]	263 cycles	—	79.3%
[2]	7530 cycles	55	97.95%
[6]	207 cycles	—	93.2%
[7]	1600 s	80	95%
[8]	2286 s	9	98%
[9]	700 cycles	8	99.1% for *S*2
[14]	80 min	8	3.4 bpm SD
[10]	357 cycles	71	97.47
[11]	326 cycles	53	91.47 and 88.95%
[12]	340 cycles	41	90.29%
[13]	—	3	2.4 bpm RMS error
this work	38.4 h	10	90.7%

Table 3 shows the estimation error bias and SD in beats per minute (bpm) for each subject with respect to the external Konica-Minolta and SomnoMedics systems. Overall, for most subjects the algorithm gives considerably good bias and SD results for a much larger dataset than that used in [14].

**Table 3:** List of value difference bias and SD between the algorithm heart rate output and those from Konica-Minolta and SomnoMedics device in bpm for each subject

Subject	Konica-Minolta		SomnoMedics	
	Bias, bpm	SD, bpm	Bias, bpm	SD, bpm
*S*01	0.15	2.3	0.15	2.3
*S*02	−0.66	3.11	−0.68	3.15
*S*03	1.4	7.62	1.4	7.62
*S*04	0.45	4.62	0.45	4.62
*S*05	2.04	7.4	2.07	7.6
*S*06	0.43	5.96	0.43	5.96
*S*07	6.77	21.42	6.77	21.42
*S*08	1.57	7.23	1.57	7.23
*S*09	−0.43	3.26	−0.43	3.26
*S*10	1.01	6.78	1.01	6.78

The sensor used for recording signals in this paper was originally designed to monitor breathing [15]. It was designed to be comfortable and easy to use. During a pilot clinical study of its use in apnoea detection, all the patients gave it a very high rating on comfort level [19]. The results in this paper show that, apart from monitoring the breathing, it is also possible to extract heart rate from the same sensor placed on the same location. This is highly advantageous for wearable health monitors since it obviates the need to use a different sensor to monitor heart rate. In other words, it could reduce the number of sensors required to be placed on patients, thus making it more comfortable for them to use in long-term monitoring. However, because the algorithm is specifically designed to work with the heart sounds obtained at the suprasternal notch, it is unlikely to perform well ‘as is’ on sounds obtained from any other location. In that sense, the algorithm is linked to the sensor location and will need to be adjusted to work with the traditional heart sounds.

Although the algorithm has been tested on a much larger dataset than any other, the number of subjects is comparatively low since this was only a pilot study to prove the feasibility of this method. Future work involves a greater clinical trial with a higher number of test subjects. Overall, the results in this Letter illustrate a strong proof of concept for heart rate monitoring using acoustic signals from the suprasternal notch. This has been demonstrated with the development of a novel heart rate extraction algorithm and its performance evaluation on a large dataset of over 38 h. The acoustic heart rate algorithm presented in this Letter also represents an advance in the field of acoustic heart rate monitoring beyond its conventional use where sensors are placed on the chest. These results will be highly useful for designers and researchers in wearable health monitoring systems by opening up the possibility of using alternative sensor locations thereby using a single sensor to monitor multiple vital signs.

## 5 Funding and declaration of interests

Conflict of interest: none declared.
